# The Novel Direct Modulatory Effects of Perampanel, an Antagonist of AMPA Receptors, on Voltage-Gated Sodium and M-type Potassium Currents

**DOI:** 10.3390/biom9100638

**Published:** 2019-10-22

**Authors:** Ming-Chi Lai, Ray-Chang Tzeng, Chin-Wei Huang, Sheng-Nan Wu

**Affiliations:** 1 Department of Pediatrics, Chi-Mei Medical Center, Tainan 710, Taiwan; vickylai621@gmail.com; 2 Department of Neurology, Tainan Municipal Hospital (Managed by Show Chwan Medical Care Corporation), Tainan 701, Taiwan; tzeng63@yahoo.com.tw; 3 Department of Neurology, National Cheng Kung University Hospital, College of Medicine, National Cheng Kung University, Tainan 701, Taiwan; 4 Institute of Basic Medical Sciences, College of Medicine, National Cheng Kung University, Tainan 701, Taiwan; 5 Department of Physiology, College of Medicine, National Cheng Kung University, Tainan 701, Taiwan

**Keywords:** perampanel, M-type K^+^ current, Na^+^ current, hippocampal cell, motor neuron, glioma cell

## Abstract

Perampanel (PER) is a selective blocker of AMPA receptors showing efficacy in treating various epileptic disorders including brain tumor-related epilepsy and also potential in treating motor neuron disease. However, besides its inhibition of AMPA-induced currents, whether PER has any other direct ionic effects in different types of neurons remains largely unknown. We investigated the effects of PER and related compounds on ionic currents in different types of cells, including hippocampal mHippoE-14 neurons, motor neuron-like NSC-34 cells and U87 glioma cells. We found that PER differentially and effectively suppressed the amplitude of voltage-gated Na^+^ currents (*I*_Na_) in mHippoE-14 cells. The IC_50_ values required to inhibit peak and late *I*_Na_ were 4.12 and 0.78 μM, respectively. PER attenuated tefluthrin-induced increases in both amplitude and deactivating time constant of *I*_Na_. Importantly, PER also inhibited the amplitude of M-type K^+^ currents (*I*_K(M)_) with an IC_50_ value of 0.92 μM. The suppression of *I*_K(M)_ was attenuated by the addition of flupirtine or ZnCl_2_ but not by L-quisqualic acid or sorafenib. Meanwhile, in cell-attached configuration, PER (3 μM) decreased the activity of M-type K^+^ channels with no change in single-channel conductance but shifting the activation curve along the voltage axis in a rightward direction. Supportively, PER suppressed *I*_K(M)_ in NSC-34 cells and *I*_Na_ in U87 glioma cells. The inhibitory effects of PER on both *I*_Na_ and *I*_K(M)_, independent of its antagonistic effect on AMPA receptors, may be responsible for its wide-spectrum of effects observed in neurological clinical practice.

## 1. Introduction

Perampanel (PER, Fycompa®) is the first approved anti-epileptic drug (AED) of the selective non-competitive antagonist of α-amino-3-hydroxy-5-methyl-4-isoxazolepropionic acid (AMPA)-type receptor class [1,2,3]. This class of AMPA-type receptors is the major subtype of ionotropic glutamate receptors. PER has been demonstrated to reduce neuronal excitation primarily through the blockade of AMPA receptors [1,2,3]. As a once-daily oral AED, it has been approved globally for the adjunctive treatment of partial epilepsy and primary generalized tonic-clonic seizures [4,5,6], although the underlying mechanism of actions through which PER exerts its broad-spectrum antiepileptic effect has not been fully elucidated.

Several studies have shown that PER can ameliorate painful sensations such as spontaneous pain symptoms [7,8,9]. PER has also been demonstrated to potentially improve post-stroke functional and cognitive impairments through multiple pathways [10,11]. In addition, PER has also been reported to be beneficial for patients with Parkinson’s disease and motor neuron diseases [12,13,14,15] and also in treating glioma-associated epilepsy [16,17,18,19]. However, several reports have shown that some of the actions of PER may not be closely linked to its blocking effect on AMPA receptors [11,20].

Molecular studies of epileptogenesis have demonstrated that specific ion channels play important roles in both genetic and acquired forms of epilepsy, particularly voltage-gated Na^+^ (Na_V_) channels [21,22,23]. Nine isoforms (Na_V_1.1-1.9) are found in mammalian excitable tissues, including the central nervous system, peripheral nervous system, skeletal muscles and heart [24]. Moreover, inhibitors of the late component of voltage-gated Na^+^ currents (*I*_Na_) such as ranolazine and eugenol have been reported to suppress seizure activity [25,26]. However, whether PER exerts any effects on the amplitude and gating of *I*_Na_ remains unclear.

Among the K^+^ channels, the KCNQ2, KCNQ3 and KCNQ5 genes are known to encode the core subunits of K_V_7.2, K_V_7.3 and K_V_7.5 channels, respectively. The enhanced activity of these voltage-gated (K_V_) channels can generate a population of K^+^ currents, namely M-type K^+^ currents (*I*_K(M)_), which display a slowly activating and deactivating property [27,28,29]. Targeting *I*_K(M)_ has been reported to be an adjunctive management strategy for various neurological disorders including epilepsy [22,29]. The involvement of *I*_K(M)_ has also been shown in motor neuron hyperexcitability in patient with motor neuron diseases [30]. Alternatively, the magnitude of delayed-rectifier K^+^ currents (e.g., K_v_2.1- or K_V_1.1-encoded current) has been demonstrated to be linked to epileptogenesis [31,32]. However, whether PER exerts any actions on these types of ionic currents remains unexplored.

Therefore, in this study, we explored the possible modulatory effects of PER on different types of ionic currents (e.g., voltage-gated Na^+^ currents [*I*_Na_], M-type K^+^ currents [*I*_K(M)_] and delayed-rectifier K^+^ currents [*I*_K(DR)_]) in hippocampal mHippoE-14 cells. We also investigated the effects of PER on *I*_K(M)_ in motor neuron-like NSC-34 cells and on *I*_Na_ in U87 glioma cells. The findings showed that PER could differentially suppress the peak and late components of *I*_Na_ in both mHippoE-14 cells and U87 glioma cells and that it could suppress *I*_K(M)_ in mHippoE-14 cells and NSC-34 cells. Therefore, in addition to its blocking effects on AMPA receptors, the actions revealed in this study may also contribute to its in vivo pharmacological actions.

## 2. Materials and Methods

### 2.1. Drugs and Solutions

Perampanel (PER, Fycompa®, 2-(2-oxo-1-phenyl-5-pyridin-2-yl-1,2-dihydropyridin-3-yl) benzonitrile, 3-(2-cyanophenyl)-5-(2-pyridyl)-1-phenyl-1,2-dihydropyridin-2-one, E2007, C_23_H_15_N_3_O) was obtained from Eisai Co., Ltd. (Tokyo, Japan). Diazoxide, 9-phenanthrol, tefluthrin, tetraethylammonium chloride (TEA) and tetrodotoxin (TTX) were purchased from Sigma-Aldrich (St. Louis, MO, USA). L-quisqualic acid (QA) and NBQX disodium salt (2,3-dioxo-6-nitro-1,2,3,4-tetrahydrobenzo[*f*]quinozaline-7-sulfonamide disodium salt) were purchased from Tocris (Bristol, UK) and gefitinib and sorafenib were obtained from Selleck (Houston, TX, USA). Tissue culture media, fetal bovine serum, L-glutamine and trypsin were obtained from Invitrogen (Carlsbad, CA, USA) and all other chemicals including CsCl, CsOH and ZnCl_2_ were regular commercial chemicals and of reagent grade. Deionized water was made using a Milli-Q water purification system (Millipore, Bedford, MA, USA).

The bath solution (i.e., HEPES-buffered normal Tyrode’s solution) used in this study was composed of 136 mM NaCl, 5.4 mM KCl, 1.8 mM CaCl_2_, 0.53 mM MgCl_2_, 5.5 mM glucose and 5.5 mM HEPES-NaOH buffer, pH 7.4. To measure macroscopic K^+^ currents (e.g., *I*_K(M)_ or *I*_K(DR)_) and to preclude contamination of Cl^-^ currents, we filled patch pipettes with solution containing 130 mM K-aspartate, 20 mM KCl, 1 mM KH_2_PO_4_, 1 mM MgCl_2_, 3 mM Na_2_ATP, 0.1 mM Na_2_GTP, 0.1 mM EGTA and 5 mM HEPES-KOH buffer, pH 7.2. To record *I*_Na_, we replaced K^+^ ions in the filling solution of the electrode with equimolar Cs^+^ ions and the pH was adjusted to 7.2 with CsOH. For measurements of whole-cell *I*_K(M)_, we used a high K^+^-bathing solution consisting of 145 mM KCl, 0.53 mM MgCl_2_ and 5 mM HEPES-KOH buffer, pH 7.4, while the recording pipette was filled with a solution containing 145 mM KCl, 2 mM MgCl_2_ and 5 mM HEPES-KOH buffer, pH 7.2. To measure the activity of single K_M_ channels, we used a pipette solution of NaCl 136.5 mM, KCl 5.4 mM, MgCl_2_ 0.53 mM and HEPES-NaOH buffer 5 mM, pH 7.4. All solutions were prepared using deionized water from a Milli-Q water purification system (APS Water Services, Inc., Van Nuys, CA). The pipette solution and culture medium were filtered on the day of use with an Acrodisc^®^ syringe filter with a 0.2 μm Supor^®^ membrane (Pall Corp., Port Washington, NY, USA).

### 2.2. Cell Preparations

Embryonic mouse hippocampal cell line (mHippoE-14, CLU198) was obtained from Cedarlane CELLutions Biosystems, Inc. (Burlington, ON, Canada) [29,33,34]. Cells were maintained at a density of 10^6^/mL in Dulbecco’s modified Eagle’s medium (DMEM) supplemented with 10% fetal bovine serum (FBS) and 2 mM L-glutamine. NSC-34 neuronal cells were kindly provided by Professor Dr. Yuh-Jyh Jong (Department of Pediatrics, Kaohsiung Medical University Hospital, Kaohsiung, Taiwan). They were grown in DMEM supplemented with 10% FBS. The glioblastoma multiforme cell line (U87) was obtained from American Type Culture Collection (ATCC, Manassas, VA) and the cells were grown in DMEM/F12 nutrient media (Invitrogen, Carlsbad, CA) supplemented with 10% FBS. The culture medium was changed every 2 to 3 days and the cells were passaged when they reached confluence. The experiments were usually performed 5 or 6 days after the cells had been cultured (60-80% confluence).

### 2.3. Electrophysiological Measurements

On the day of the measurements, mHippoE-14, NSC-34 or U78 cells were dissociated and an aliquot of cell suspension was immediately transferred to a custom-made recording chamber affixed to the stage of a DM-IL inverted microscope (Leica, Wetzlar, Germany). To visualize changes in cell size during the recordings, the microscope was coupled to a digital video system (DCR-TRV30; Sony, Japan) with a magnification of up to 1500×. The cells were bathed at room temperature (20–25 °C) in normal Tyrode’s solution, the composition of which is described above. The patch electrodes were fabricated with Kimax-51 capillaries with an outside diameter of 1.5 to 2.0 mm (#34500; Kimble, Vineland, NJ) using either a vertical PP-83 puller (Narishige, Tokyo, Japan) or a horizontal P-97 Flaming/Brown micropipette puller (Sutter, Novato, CA) and their tips were fire polished with an MF-83 microforge (Narishige). The electrodes used for the recordings had a tip resistance of 3-5 MΩ when filled with different internal solutions as described above. Ion currents were measured in whole-cell or cell-attached configurations of the standard patch-clamp technique with either an RK-400 (Bio-Logic, Claix, France) or an Axopatch-200B (Molecular Devices, Sunnyvale, CA, USA) amplifier [35]. All potentials were offset for liquid junction potentials which arose at the electrode tip when the composition of the pipette solution was different from that in the bath. Single K_M_-channel activity measured from the mHippoE-14 cells was analyzed using pCLAMP 10.7 software (Molecular Devices).

### 2.4. Data Recordings

Data comprising both potential and current traces were stored online in an Acer SPIN-5 touchscreen laptop computer (SP513-52N-55WE; Taipe, Taiwan) at 10 kHz equipped with a Digidata 1440A interface (Molecular Devices, Inc., Sunnyvale, CA), which was used for analog-to-digital/digital-to-analog conversion. During the recordings, the latter device was controlled by pCLAMP 10.7 software (Molecular Devices) run under Windows 10 (Redmond, WA, USA) and the signals were simultaneously monitored on an LCD monitor (MB169B+; ASUS, Taipei, Taiwan) through a USB type-C connection. Current signals were low-pass filtered at 2 kHz with a FL-4 four-pole Bessel filter (Dagan, Minneapolis, MN, USA) to minimize background noise. Through digital-to-analog conversion, various pCLAMP-generated voltage-clamp profiles with different waveforms were applied to establish the current-voltage (*I-V*) relationship of *I*_K(M)_ or *I*_K(DR)_. As high-frequency stimuli were needed, an Astro-med Grass S88X dual output pulse stimulator (Grass Technologies, West Warwick, RI, USA) was used. 

### 2.5. Data Analysis

The digital signals were examined and analyzed offline using either pCLAMP 10.7 (Molecular Devices), 64-bit OriginPro 2016 (OriginLab, Northampton, MA, USA) or custom-made macros created in Microsoft Excel^®^ 2013 which was run under Windows 10 (Redmond, WA, USA). The concentration-response data for the inhibition of either peak and late *I*_Na_, *I*_K(M)_ or *I*_K(DR)_ in the mHippoE-14 cells were least-squares fitted to the Hill equation as follows:

(1) $$percentage\;inhibition=\frac{E_{max}\times {\left[PER\right]}^{n_{H}}}{{\left[PER\right\}}^{n_{H}}+IC_{50}^{n_{H}}}$$

where [PER] is the PER concentration, IC_50_ and n_H_ are the concentrations required for a 50% inhibition and the Hill coefficient, respectively and E_max_ is the maximal suppression of both peak or late *I*_Na_ and *I*_K(M)_ caused by the presence of PER.

### 2.6. Single-Channel Analysis

Single K_M_-channel currents recorded from mHippoE-14 neurons were analyzed using pCLAMP 10.7. Single-channel amplitudes measured with or without the addition of PER were determined by fitting Gaussian distributions to the amplitude histograms of the closed and open states. The single-channel conductance of K_M_ channels was estimated using linear regression with averaged values of single-channel amplitudes measured at different levels of membrane potentials relative to the bath. To determine the voltage dependence of the inhibitory effect of PER on the activity of K_M_ channels, the patch obtained with or without the addition of PER was held at different membrane potentials. The activation curves of K_M_-channel openings in the absence and presence of PER (3 μM) were appropriately fitted by the Boltzmann equation:

(2) $$Relative\;open\;probability=\frac{P_{max}}{1+e^{\left[\frac{-\left(V-V_{1/2}\right)qF}{RT}\right]}}$$

where P_max_ is the maximal probability of K_M_-channel openings in the control (i.e., in the absence of PER) maintained at +70 mV relative to the bath, V_1/2_ is the voltage at which half-maximal activation of K_M_ channels occurs, q is the apparent gating charge, F is Faraday’s constant, R is the universal gas constant, T is absolution temperature and F/RT = 0.04 mV^−1^.

### 2.7. Statistical Analysis

The data were analyzed and expressed as mean ± standard error of the mean (SEM). The paired or unpaired Student’s *t*-test or one-way analysis of variance (ANOVA) followed by post-hoc Fisher’s least-significance difference test for multiple-group comparisons were used for statistical evaluations of the differences among means. We used the non-parametric Kruskal-Wallis test, as the assumption of normality underlying ANOVA was violated. Statistical analyses were performed using IBM SPSS^®^ version 20.0 (IBM Corp., Armonk, NY, USA). *p* < 0.05 was considered to be statistically significant.

## 3. Results

### 3.1. Inhibitory Effect of PER on Voltage-Gated Na^+^ Current (I_na_) in Hippocampal Mhippoe-14 Cells

**Figure 1:**
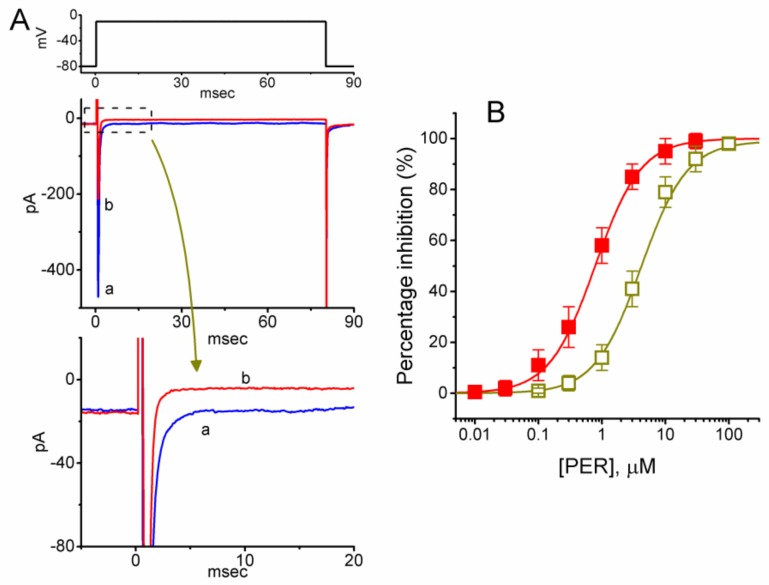
Inhibitory effects of perampanel (PER) on the peak and late components of voltage-gated Na+ current (INa) in mouse hippocampal mHippoE-14 cells. In these experiments, we immersed the cells in Ca^2+^-free Tyrode’s solution containing 10 mM tetraethylammonium chloride (TEA) and the recording pipette was filled with a Cs+-containing solution. Once the whole-cell mode had been firmly established, the voltage protocol from −80 mV to different voltages with a duration of 80 msec was applied at a rate of 0.5 Hz. (**A**) Superimposed INa traces obtained in the controls (a) and presence (b) of 3 μM PER. The uppermost part shows the voltage protocol used. The lower part depicts an expanded record of late INa (INa,L) as indicated in the dashed box. (**B**) Concentration-response relationships for PER-induced suppression of INa measured at the beginning (□) and end (■) of the depolarizing pulses (mean ± SEM; *n* = 13 for each point). Smooth lines represent the least-squares fit to a modified Hill function as described in the Materials and Methods.

Although PER is a known antagonist of AMPA receptors, little is known regarding whether PER exerts any effects on ionic currents other than its antagonistic effect on AMPA-induced currents or Ca^2+^ rise. Therefore, in the initial set of experiments, cells were bathed in Ca^2+^-free Tyrode’s solution containing 10 mM tetraethylammonium chloride (TEA) and the recording pipette was loaded with a Cs^+^-containing solution. As shown in Figure 1, upon membrane depolarization from -80 to -10 mV, the whole-cell *I*_Na_, which comprised the peak and late components of the current, was readily elicited in these cells. In particular, the addition of PER progressively and differentially suppressed peak and late *I*_Na_ in a concentration-dependent manner. For example, when mHippoE-14 cells were rapidly depolarized from −80 to −10 mV, the addition of PER (3 μM) decreased the peak amplitude of *I*_Na_ (i.e., at the beginning of the depolarizing pulse) from 469 ± 22 to 226 ± 26 pA (*n* = 13, *p* < 0.05); however, it also reduced the late component of *I*_Na_ (i.e., at the end of the depolarizing pulse) from 13.7 ± 2.7 to 4.2 ± 1.1 pA (n = 13, *p* < 0.05). After washout of the compound, the amplitudes of peak and late *I*_Na_ returned to 452 ± 21 and 12.3 ± 2.3 pA (*n* = 12, *p* < 0.05), respectively. 

The relationships between PER concentration and the percentages of inhibition of the peak and late components of *I*_Na_ are illustrated in Figure 1B. The IC_50_ values required for the inhibitory effects of PER on peak and late *I*_Na_ were 4.12 and 0.78 μM, respectively. Therefore, PER exerted a significant action on the inhibition of peak and late *I*_Na_ in a concentration-dependent manner. More specifically, during cell exposure to PER, late *I*_Na_ in response to rapid depolarization was suppressed to a greater extent than peak *I*_Na_.

### 3.2. The presence of Tefluthrin (Tef) and Tef Plus PER on I_na_ in Mhippoe-14 Cells

**Figure 2:**
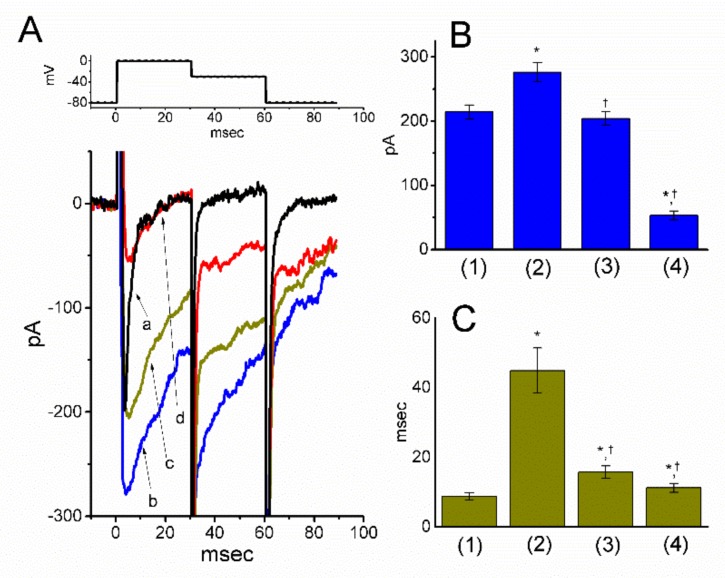
Effects of tefluthrin (Tef) and Tef plus PER on INa in mHippoE-14 cells. These experiments used the same protocol as described above. Tef (10 μM) was added to the bath and in the continued presence of Tef, PER was subsequently applied to the investigated cells at different concentrations. (**A**) Original INa traces obtained in the control (**a**) or in the presence of 10 μM Tef alone (**b**), 10 μM Tef plus 1 μM PER (**c**) and 10 μM Tef plus 3 μM PER (**d**). The upper part indicates the voltage-step protocol used; that is, the examined cells were depolarized from −80 to 0 mV for 30 msec, then repolarized to −30 mV. (**B**) and (**C**), respectively, depict the summary bar graphs indicating the peak amplitude and deactivation time constant (τdeact) of depolarization-induced INa obtained in the controls and during the exposure to Tef and Tef plus PER (mean ± SEM; *n* = 12 for each point). (1): controls (in the absence of Tef or PER); (2): 10 μM Tef alone; (3): 10 μM Tef plus 1 μM PER; (4): 10 μM Tef plus 3 μM PER. * Significantly different from the controls (i.e., bars (1)) (*p* < 0.05) and †significantly different from the 10 μM Tef alone group (i.e., bars (2)) (*p* < 0.05).

Tef, a type-I pyrethroid, has been demonstrated to activate *I*_Na_ [25,36,37]. We next explored whether in the continued presence of Tef, the subsequent addition of PER could suppress depolarization-induced *I*_Na_. As shown in Figure 2A,B, consistent with previous observations [25,36], during cell exposure to 10 μM Tef, the peak amplitude of depolarization-elicited *I*_Na_ was progressively increased along with an increase in deactivating time constant (τ_deact_) measured at the level of −30 mV. Of note, the subsequent addition of PER (3 μM), still in the presence of 10 μM Tef, was effective both at suppressing Tef-stimulation of *I*_Na_ and at reversing its increase in τ_deact_. For example, the addition of Tef (10 μM) increased peak *I*_Na_ from 214 ± 11 to 276 ± 15 pA (*n* = 12, *p* < 0.05) and prolonged the slow component of τ_deact_ from 8.7 ± 1.1 to 44.9 ± 6.5 msec (*n* = 12, *p* < 0.05). In the continued presence of Tef, the further addition of 3 μM PER significantly decreased peak *I*_Na_ and τ_deact_ to 53 ± 7 pA and 11.2 ± 1.3 msec (*n* = 12, *p* < 0.05), respectively. Therefore, despite the presence of an *I*_Na_ stimulator (e.g., Tef), PER still remained effective at suppressing peak and late *I*_Na_ in mHippoE-14 cells.

### 3.3. Effect of PER on M-type K^+^ Currents (I_K(M)_) in Mhippoe-14 Cells

**Figure 3:**
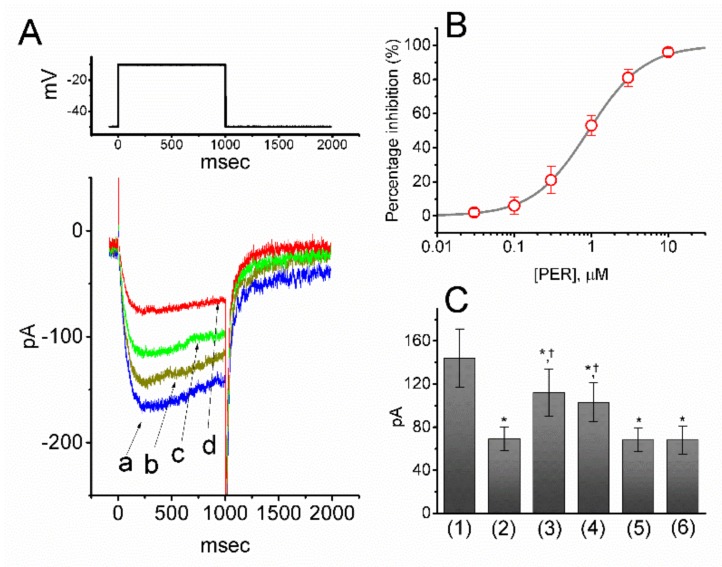

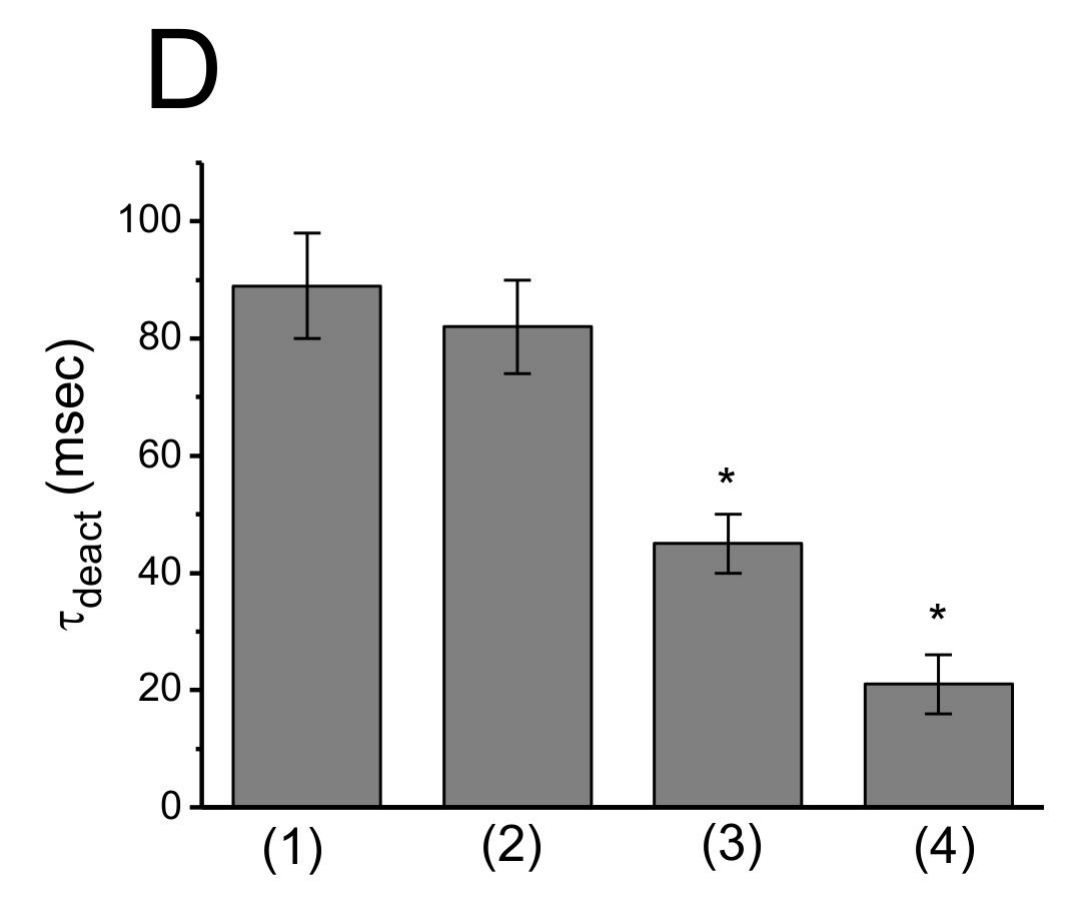
Concentration-dependent effect of PER on M-type K^+^ current (IK(M)) in mHippoE-14 cells. In these experiments, the cells were immersed in high-K^+^, Ca^2+^-free Tyrode’s solution and each pipette was filled with K^+^-containing solution. (**A**) Original IK(M) traces elicited by a long-lasting membrane depolarization from −50 to −10 mV (indicated in the upper part of (**A**)). a: controls; b: 0.1 μM PER; c: 0.3 μM PER; d: 1 μM PER. (**B**) Concentration-response relation for PER-induced inhibition of IK(M). Each point represents the mean ± SEM (*n* = 12–14). The smooth line indicates the best fit to the Hill equation. The IC50 value, the maximally inhibited percentage of the current and Hill coefficient were 0.92 μM, 100% and 1.2, respectively. (**C**) Summary bar graph showing the effects of PER, PER plus flupirtine, PER plus ZnCl2, PER plus L-quisqualic acid (QA) and PER plus sorafenib on IK(M) amplitude (mean ± SEM; *n* = 11 for each bar). Current amplitude was measured at the end of each depolarizing step. (1): control; (2): 1 μM PER; (3): 1 μM PER plus 10 μM flupirtine; (4): 1 μM PER plus 10 μM ZnCl2; (5) 1 μM PER plus 10 μM L-quisqualic acid (QA); (6) 1 μM PER plus 10 μM sorafenib. * Significantly different from the control (i.e., bar (1)) (*p* < 0.05) and Ϯsignificantly different from the 1 μM PER alone group (i.e., bar (2)) (*p* < 0.05). (**D**) Summary bar graph showing the effect of PER (0.1, 0.3 and 1 µM) on the τ_deact_ value of IK(M) (mean ± SEM; *n* = 9 for each bar). * Significantly different from the control (*p* < 0.05). (1): control; (2) 0.1 µM PER; (3) 0.3 µM PER; (4) 1 µM PER.

To elucidate whether PER can perturb other types of ionic currents in neurons, we further studied its effects on *I*_K(M)_ in response to long-lasting depolarizing pulses. To measure *I*_K(M)_, cells were bathed in high-K^+^, Ca^2+^-free solution and the recording pipette was filled with K^+^-containing solution. Once the whole-cell mode had been established, voltage pulses from −50 to −10 mV with a duration of 1 sec were delivered to the cells and an *I*_K(M)_ was readily evoked [29,38]. Of note, as the cells were exposed to increasing concentrations of PER, the *I*_K(M)_ amplitude in response to membrane depolarization progressively decreased (Figure 3A,B). For example, the addition of PER (1 μM) decreased the current amplitude at the end of the depolarizing pulse from 141 ± 11 to 67 ± 9 pA (*n* = 12, *p* < 0.05). After washout of PER, the current amplitude returned to 137 ± 11 pA (*n* = 11, *p* < 0.05). Likewise, the presence of PER also decreased the τ_deact_ value of *I*_K(M)_ obtained upon return to −50 mV (Figure 3D). For example, the τ_deact_ of *I*_K(M)_ obtained under our experimental conditions was also significantly decreased from 89 ± 9 to 21 ± 5 msec (*n* = 12, *p* < 0.05) in the presence of PER (1 μM). As illustrated in Figure 3B, PER (0.03–10 μM) effectively suppressed the *I*_K(M)_ amplitude in a concentration-dependent manner. From the Hill equation, the values of IC_50_ and Hill coefficient for PER-inhibited *I*_K(M)_ were estimated to be 0.92 μM and 1.2, respectively. These results clearly showed that, in addition to the suppression of both peak and late components of *I*_Na_, the presence of PER per se was effective at suppressing the amplitude of *I*_K(M)_ in mHippoE-14 cells. However, NBQX disodium salt (10 µM), another antagonist of AMPA receptors, did not have any effect on *I*_K(M)_ amplitude (143 ± 12 pA [in the control] versus 142 ± 13 pA [in the presence of 10 µM NBQX disodium salt], *n* = 8, *p* > 0.05). Of note and unexpectedly, as the cells were constantly exposed to PER, the subsequent addition of either flupirtine (10 μM) or ZnCl_2_ (10 μM) but not L-quisqualic acid (QA; 10 μM) or sorafenib (10 μM), significantly reversed the PER-mediated suppression of *I*_K(M)_ (Figure 3C). Flupirtine and ZnCl_2_ have both been reported to activate *I*_K(M)_ [29,39,40], QA is a known agonist of AMPA receptors [41] and sorafenib can suppress the activity of tyrosine kinases. Moreover, in mHippoE-14 cells which were pretreated with NBQX (10 µM) for 6 h, the inhibition by PER (1 µM) of *I*_K(M)_ amplitude still remained efficacious, as evidenced by a significant reduction of the current from 146 ± 13 to 69 ± 11 pA (*n* = 11, *p* < 0.05). In keeping with these results, the presence of PER (1 µM) also decreased the τ_deact_ value of the current. As cells were preincubated with NBQX, the activity of endogenous glutamate concentrations released from these cells could have been fully removed. Therefore, the PER-mediated suppression of *I*_K(M)_ observed in these cells was not associated with either its antagonistic effect on AMPA receptors or changes in the activity of tyrosine kinases.

### 3.4. Suppressive Effect of PER on The Activity of M-Type K^+^ (K_M_) Channels In Mhippoe-14 Cells

**Figure 4:**
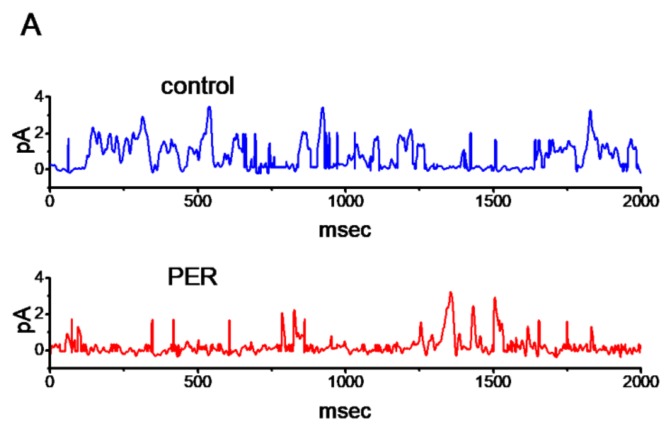

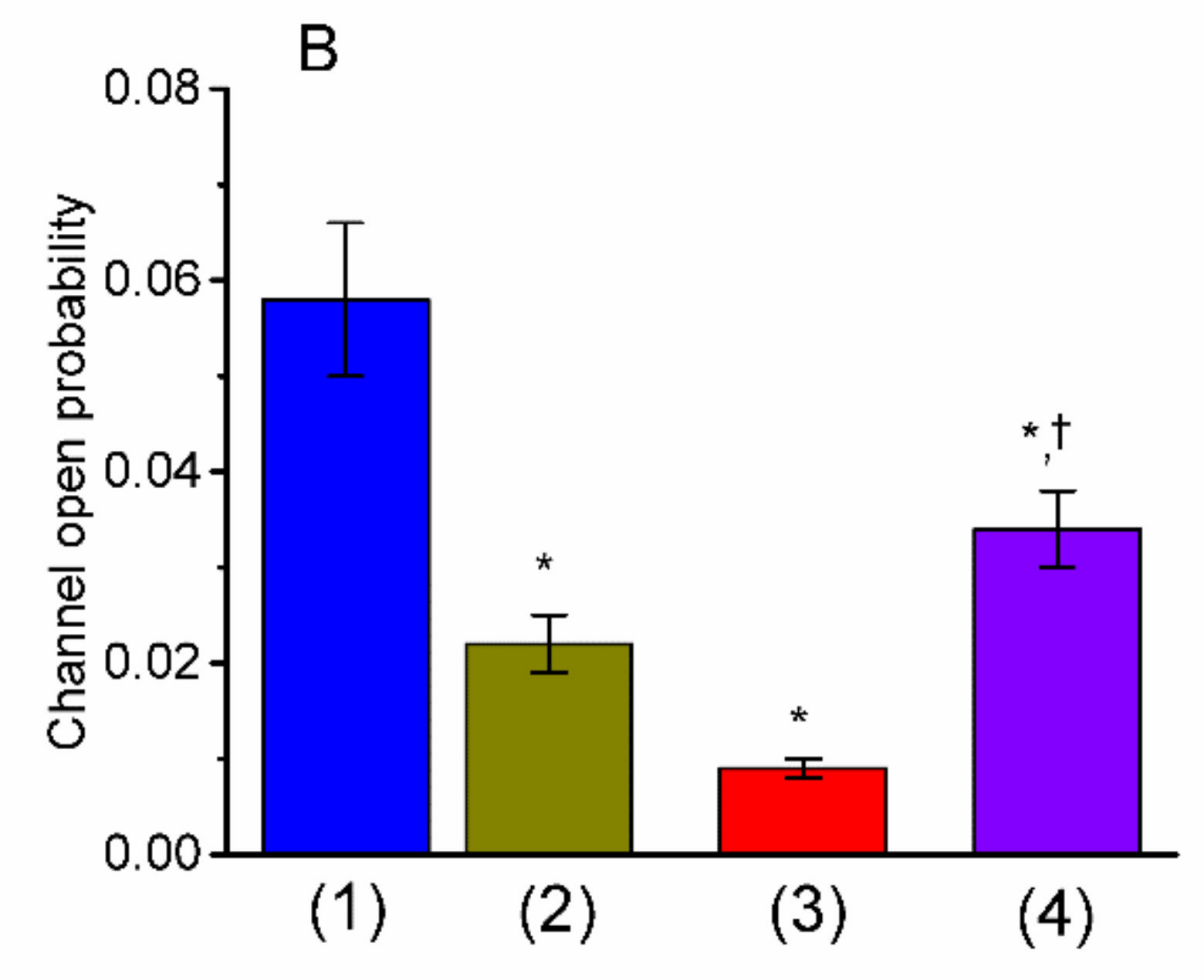
Effect of PER on the activity of M-type K^+^ (KM) channels recorded from mHippoE-14 cells. In these cell-attached single-channel recordings, the cells were bathed in high-K^+^, Ca^2+^-free solution, the recording pipette was filled with low-K^+^ (5.4 mM) solution and the potential was held at +30 mV relative to the bath. (**A**) Superimposed KM-channel traces in the absence (upper) and presence (lower) of 1 μM PER. Of note, the opening events caused the upper deflections of the trace in this on-cell patch. (**B**) Summary bar graph showing the effects of PER and PER plus flupirtine on the probabilities of KM channels that would be open (mean ± SEM; *n* = 11 for each bar). (1): controls; (2): 1 μM PER; (3): 3 μM PER; (4) 3 μM PER plus 10 μM flupirtine. * Significantly different from the controls (*p* < 0.05) and Ϯsignificantly different from the PER (3 μM) alone group (*p* < 0.05).

The PER-mediated inhibition of *I*_K(M)_ described above may have arisen from its effects on the activity or gating of K_M_ channels. To further determine the depressant action of PER on whole-cell *I*_K(M)_, we further explored how PER influenced the activity of single K_M_-channel currents in these cells. In this set of single-channel current recordings, we bathed the cells in high-K^+^, Ca^2+^-free solution and the filling solution contained low-K^+^ solution, the composition of which is described above. As shown in Figure 4, when the potential was maintained at +30 mV relative to the bath, the activity of K_M_ channels, which underwent open-closed transitions, was readily detected. As PER was applied to the bath, the probability of K_M_-channel opening was significantly decreased. For example, the addition of PER (3 μM) significantly reduced the channel activity from 0.058 ± 0.008 to 0.009 ± 0.001 (*n* = 11, *p* < 0.05). The further addition of flupirtine (10 μM), still in the presence of PER, was effective at increasing the K_M_-channel activity to 0.034 ± 0.004 (n=11, *P*<0.05). In addition, the mean open time of the channel in the presence of 3 μM PER was significantly decreased to 2.3 ± 0.3 msec (*n* = 11, *p* < 0.05) from a control time of 4.5 ± 0.8 msec (n=11). 

### 3.5. Effect of PER on I-V Relationships with K_M_ Channels

**Figure 5:**
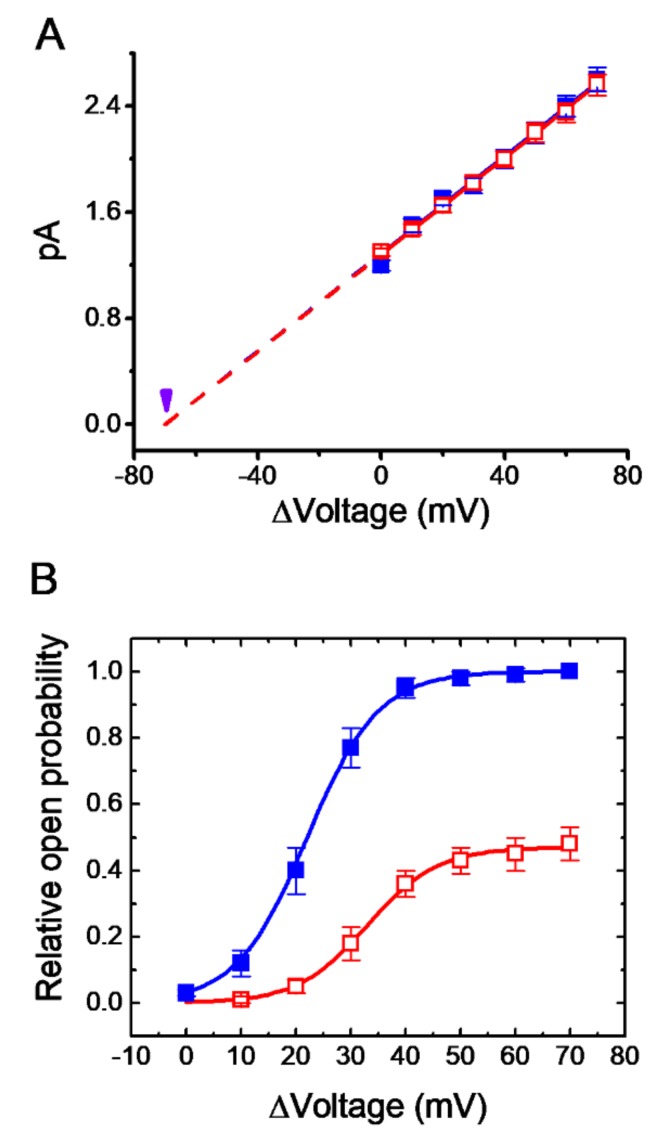
Effect of PER on current-voltage (I-V) relationships and voltage-dependent activation of KM channels in mHippoE-14 cells. In these single-channel recordings, the cells were bathed in high K^+^, Ca^2+^-free solution and the recording pipette was filled with low-K^+^ solution. (**A**) The I-V curves of KM channels in the absence (■) and presence (□) of 3 μM PER (mean ± SEM; *n* = 11 for each point). Each dashed line is pointed toward the level of the resting potential (i.e., −70 mV which is indicated by the arrowhead). Of note, the single-channel conductance (i.e. I-V relationship) of the channel in the controls was virtually overlaid with that during exposure to PER (3 μM). (**B**) The activation curve (i.e., relative channel open probability versus Δvoltage) of KM channels with or without the addition of 3 μM PER (mean ± SEM; *n* = 11 for each point). The smooth curve was least-squares fit to a Boltzmann function as described in the Materials and Methods. ■: controls; □: in the presence of 3 μM PER.

We then investigated the effect of PER on the activity of K_M_ channels at different levels of membrane potential. The single-channel amplitudes increased with greater depolarization and the presence of PER decreased the probability of K_M_-channel openings at different levels of membrane potential (Figure 5A). However, the single-channel conductance calculated from linear *I-V* relationships between the absence and presence of PER (3 μM) was only slightly perturbed (18.4 ± 0.9 pS [in the absence of PER] versus 18.3 ± 0.9 pS [in the presence of 3 μM PER], *n* = 11, *p* > 0.05). These results strongly indicated that despite its inhibitory effects on the probability of K_M_ channel opening, PER exerted little or no effect on single-channel conductance of these channels recorded from mHippoE-14 cells.

### 3.6. Rightward Shift of the Activation Curve of K_M_ Channels Caused by PER

The voltage dependence of K_M_ channels was further studied in the presence of PER. Figure 5B shows the activation curve of K_M_ channels with or without the application of PER (3 μM). The plots of the relative open probability of K_M_ channels as a function of the potential relative to the bath were least-squares fit with a Boltzmann function as described in the Materials and Methods. For the control, P_max_ = 0.99 ± 0.01, V_1/2_ = 22 ± 4 mV, q = 6.5 ± 0.9 e (*n* = 11), whereas in the presence of 3 μM PER, P_max_ = 0.48 ± 0.06, V_1/2_ = 33 ± 5 mV, q =6.4 ± 0.8 (*n* = 11). Therefore, the addition of PER caused an approximately 11-mV right shift along the voltage axis in the activation curve of K_M_ channels; however, there was no significant modification of the gating charge (i.e., q) of the K_M_ channels in its presence. These results clearly showed that the extent of the changes in K_M_-channel activity caused by PER was dependent on the level of membrane potential and that besides its reduction in the maximal probability of K_M_-channel opening, the addition of PER could modify the voltage dependence of K_M_ channels.

### 3.7. Effect of PER on Delayed-Rectifier K^+^ Currents (I_K(DR)_) in Mhippoe-14 Cells

**Figure 6:**
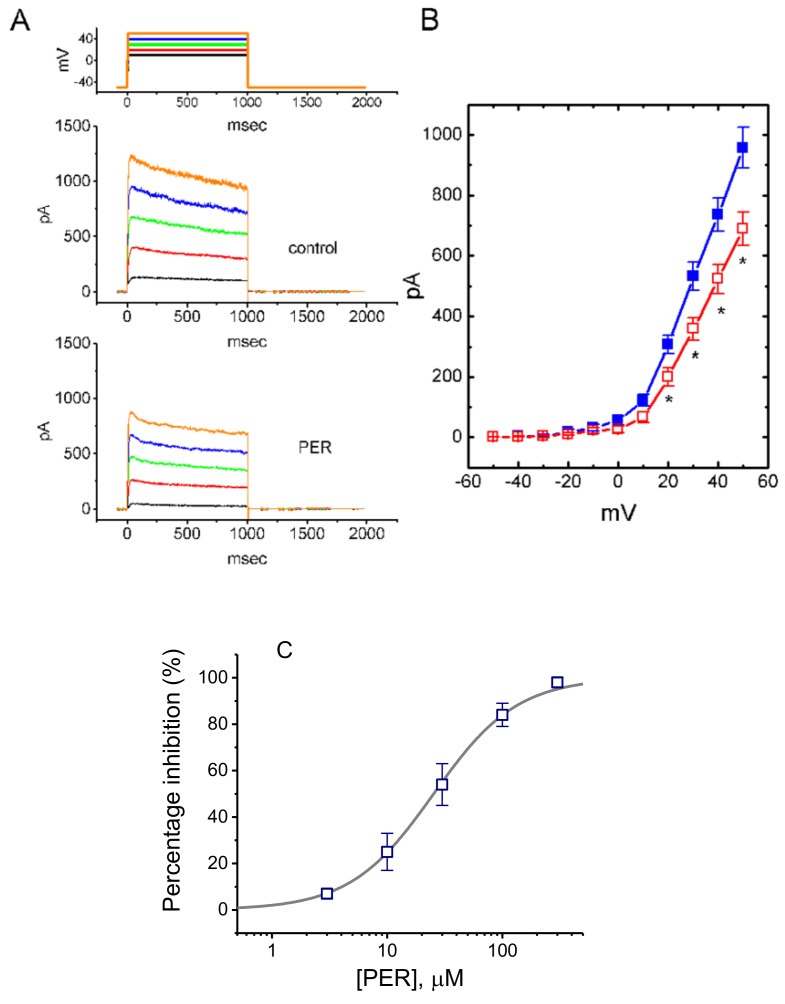
Effect of PER on delayed-rectifier K^+^ current (IK(DR)) in mHippoE-14 cells. The whole-cell current recordings were conducted in cells bathed in Ca^2+^-free Tyrode’s solution containing 1 μM tetrodotoxin (TTX). (**A**) Superimposed current traces in the absence (upper) and presence (lower) of 10 μM PER. The upper part indicates the voltage protocol applied. (**B**) Averaged I-V relationships of IK(DR) obtained in the controls (■) and during exposure to 10 μM PER (□) (mean ± SEM; *n* = 11 for each point). * Significantly different from the controls at the same level of membrane potential (*p* < 0.05). (**C**) Concentration-response curve for PER-induced inhibition of IK(DR) in mHippoE-14 cells. The examined cells were depolarized from −50 to +50 mV with a duration of 1 s and current amplitudes was measured at the end of depolarizing pulses during exposure to different PER concentrations was compared with the control value (mean ± SEM; *n* = 7–9 for each point). The continuous line represents the best fit to a Hill function. The values for IC50, maximally inhibited percentage of IK(DR) and the Hill coefficient were 25.3 µM, 100% and 1.2, respectively.

We also studied whether the presence of PER could modify delay-rectifier K^+^ currents (*I*_K(DR)_) in mHippoE-14 cells. This set of experiments was conducted with the cells bathed in Ca^2+^-free Tyrode’s solution containing 1 μM tetrodotoxin (TTX) and the recording pipette filled with K^+^-containing solution. The addition of PER at a concentration of 3 μM had little or no effect on *I*_K(DR)_ elicited in response to membrane depolarization from −50 to +50 mV. As shown in Figure 6A,B, the *I*_K(DR)_ examined at different depolarizing steps was suppressed by cell exposure to PER (10 μM). For example, on step depolarization from −50 to +50 mV, PER (10 μM) significantly decreased the amplitude of *I*_K(DR)_ from 956 ± 68 to 689 ± 55 pA (*n* = 11, *p* < 0.05). However, the time course of *I*_K(DR)_ inactivation in response to such long-lasting membrane depolarization was not modified in the presence of PER. Figure 6B illustrates the *I-V* relationships of *I*_K(DR)_ measured at the end of each depolarizing pulse in the controls and during cell exposure to 10 μM PER. 

The relationship between the PER concentration and the percentage inhibition of *I*_K(DR)_ was determined and then constructed. As illustrated in Figure 6C, PER suppressed *I*_K(DR)_ in a concentration-dependent manner with an IC_50_ value of 25.3 µM. Therefore, distinct from either *I*_Na_ or *I*_K(M)_, the *I*_K(DR)_ inherently in mHippoE-14 cells tended to be relatively resistant to suppression by PER. 

### 3.8. Effect of PER on I_K(M)_ in Motor Neuron-Like NSC-34 Cells

**Figure 7:**
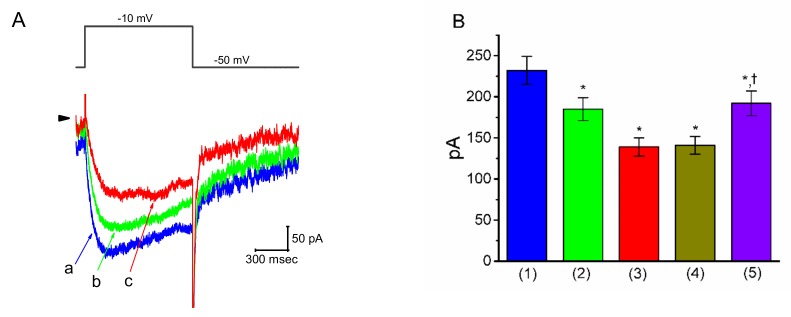
Effect of PER on IK(M) in motor neuron-like NSC-34 cells. In this set of experiments, the cells were bathed in high-K^+^, Ca^2+^-free solution and the recording pipette was filled with K^+^-containing solution. (**A**) Superimposed IK(M) traces obtained in the controls (**a**) and during cell exposure to 0.3 µM PER (**b**) and 1 μM PER (**c**). The upper part indicates the step protocol applied. (**B**) Summary bar graph showing the effects of PER, PER plus 9-phenanthrol and PER plus ZnCl_2_ on IK(M) amplitude (mean ± SEM; *n* = 12 for each bar). IK(M) amplitude was measured at the end of each depolarizing step. (1): controls; (2): 0.3 μM PER; (3): 1 μM PER; (4): 1 μM PER plus 3 μM 9-phenanthrol; (5): 1 μM PER plus 10 μM ZnCl2. * Significantly different from the controls (*p* < 0.05) and †significantly different from the PER (1 μM) alone group (*p* < 0.05).

Earlier studies have demonstrated the effectiveness of PER in treating Parkinson’s disease and motor neuron diseases [1,12,13,14,15]. We thus wanted to explore whether the presence of PER could produce any effects on *I*_K(M)_ in other types of central neurons (e.g., motor neuron-like NSC-34 cells). As shown in Figure 7A,B, under the same experimental conditions used for the mHippoE-14 cells, the depolarizing step from −50 to −10 mV elicited an *I*_K(M)_ in NSC cells bathed in high-K^+^, Ca^2+^-free solution [38]. As the cells were exposed to different concentrations of PER, the amplitude of *I*_K(M)_ in response to the long-lasting depolarizing step progressively decreased. In the continued presence of 1 μM PER, the subsequent addition of ZnCl_2_ (10 μM) was effective at reversing the PER-mediated suppression of *I*_K(M)_; however, 9-phenanthrol, an activator of intermediate-conductance Ca^2+^-activated K^+^ (IK_Ca_) channels [29], had a minimal effect (Figure 7B). These results indicated that the magnitude of PER-induced block of *I*_K(M)_ in NSC-34 cells was very similar to that in the mHippoE-14 cells described above.

### 3.9. Effect of PER on I_Na_ in U87 Glioma Cells

**Figure 8:**
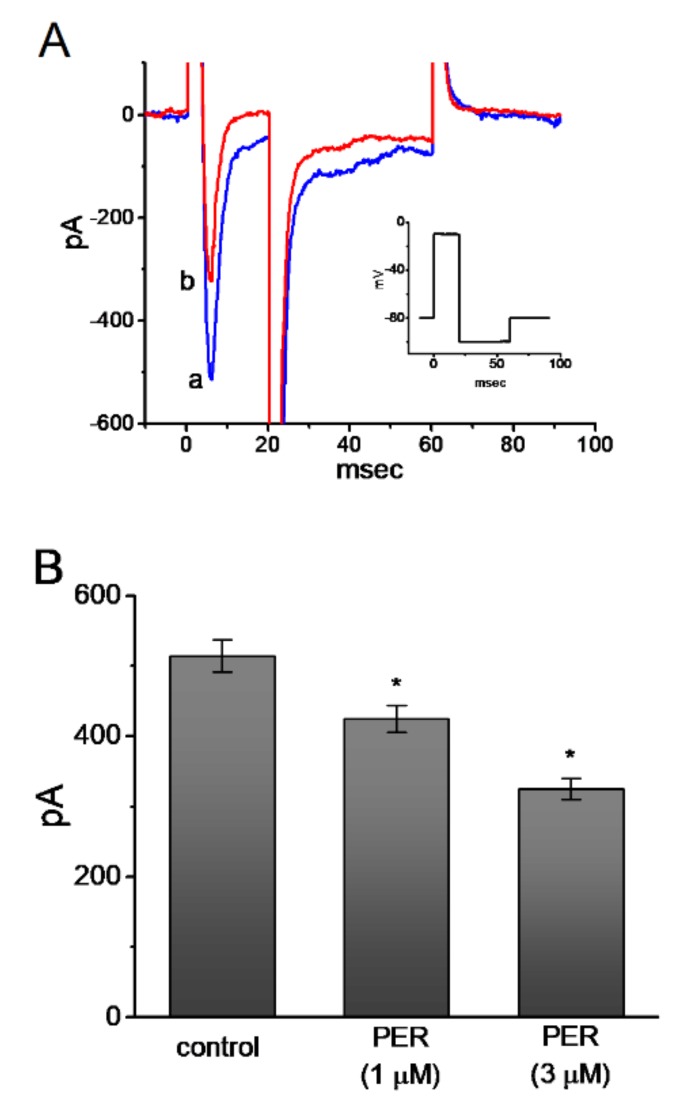
Inhibitory effect of PER on INa in U87 glioma cells. The experimental protocol used was similar to that described above for mHippoE-14 cells. (**A**) Superimposed INa traces obtained in the controls (a) and during cell exposure to 3 μM PER (b). Inset indicates the voltage-clamp protocol used. (**B**) Summary bar graph showing the inhibitory effects of 1 or 3 μM PER on peak amplitude of INa in response to the rapid depolarizing step (mean ± SEM; *n* = 11 for each bar). * Significantly different from the controls (*p* < 0.05).

It has recently been demonstrated that PER can effectively suppress the epileptic activity associated with glioma [16,17,18,19]. Na_V_ channels are also functionally expressed in electrically non-excitable cells including glial cells [42]. Therefore, we conducted a final set of whole-cell experiments with U87 glioma cells to determine whether PER can perturb any effects on ion currents, particularly on *I*_Na_. The cells were bathed in Ca^2+^-free Tyrode’s solution and the pipette was filled with Cs^+^-containing solution and the rapid depolarizing step from −80 to −10 mV evoked *I*_Na_. As shown in Figure 8A and 8B, the peak and late components of *I*_Na_ in these glioma cells diminished during exposure to 1 and 3 μM PER. The Na_V_ channels could be functionally expressed in U87 glioma cells and could create the expressed *I*_Na_ phenotype [42]. More importantly, similar to that described above in mHippoE-14 cells, *I*_Na_ recorded from glioma cells could still be suppressed by the presence of PER. The deactivating time course of the current measured at the level of −100 mV was also significantly shortened in its presence.

## 4. Discussion

The results of the present study demonstrate that in hippocampal mHippoE-14 cells, PER is capable of producing a depressant action on *I*_Na_ in a concentration- and time-dependent manner. PER preferentially suppressed the late over peak component of *I*_Na_ (IC_50_ value = 0.78 versus 4.12 μM), with a 5.3-fold selectivity for its suppression of late versus peak *I*_Na_. Both ranolazine and eugenol, which are inhibitors of late *I*_Na_, have previously been shown to suppress seizure activity [25,26]. Therefore, in addition to blockade of AMPA receptors, PER-induced block of *I*_Na_ may be another mechanism by which it depresses the excitability of neurons in vivo [22].

Several studies on healthy male volunteers who received 14 days of PER treatment reported blood PER levels ranging from 212 to 358 ng/mL (0.61–1.01 μM) with a 4-mg daily dose to 275 to 456 ng/mL (0.79–1.31 μM) with a 6-mg daily dose [43,44]. In addition, assuming 95% protein binding, free plasma PER concentrations ranged between 0.03 and 0.07 μM. However, at efficacious doses, PER concentrations at the receptors would be much lower than the IC_50_ value for the inhibition of neuronal transmission in hippocampal slices but still within the range of inhibitory effects on AMPA receptor-mediated responses [44]. Therefore, the concentration ranges used for the PER-mediated inhibition of peak and late *I*_Na_ or *I*_K(M)_ appear to be clinically achievable.

Most *I*_K(M)_ inherently in neurons is created by heteromultimers of K_V_7.2 and K_V_7.3. These channels have slow activation and deactivation kinetics, a relatively negative threshold for activation and little inactivation under physiological conditions [27,28,29]. Due to these distinctive features, K_M_-channel activity has a strong control over neuronal excitability. Thus, their negative threshold for activation allows a small number of K_M_ channels to be open near the resting membrane potential, conferring strong control over the threshold for action potential firing. In turn, their slow gating kinetics and the absence of inactivation provide a mechanism for control over action potential firing (e.g., accommodation of firing rate). In the current study, the IC_50_ value for PER-mediated suppression of *I*_K(M)_ was 0.92 μM, a value which is clinically achievable. 

Moreover, the inhibition of *I*_Na_ could be indirectly altered by the PER-induced inhibition of *I*_K(M)_, as the suppression of *I*_Na_ might be exacerbated by *I*_K(M)_ inhibition. In this study, the presence of PER produced a rightward shift along the voltage axis in the activation curve of K_M_ channels with no change in the gating charge of channel activation. As such, the PER molecule is capable of interacting with single K_M_ channels in a voltage-dependent fashion and its inhibitory action would be dependent on the pre-existing level of resting potential, the PER concentration achieved or both. Therefore, it is conceivable that, at clinically relevant concentrations, PER influences the availability of ion channels responsible for maintaining the high electrical excitability of neurons.

In this study, in the continued presence of PER, the subsequent addition of either flupirtine or ZnCl_2_ reversed the PER-induced suppression of *I*_K(M)_. However, neither 9-phenanthrol, an activator of IK_Ca_ channels [29], nor diazoxide, known to activate ATP-sensitive K^+^ channels, produced any effect. Consequently, the PER-induced decrease in *I*_K(M)_ observed in this study seems unlikely to be predominantly linked to changes in the activity of IK_Ca_ or ATP-sensitive K^+^ channels, although these two types of ionic channels may be functionally active in central neurons [29,34,45].

Recent studies have linked the activity of tyrosine kinases to the deregulated trafficking of AMPA receptors in Huntington’s disease models [46]. However, in our study, as mHippoE-14 cells were continually exposed to PER, the subsequent addition of gefitinib or sorafenib, which are known to inhibit tyrosine kinases, did not exert any effects on the reduction of *I*_K(M)_. Therefore, the PER-induced suppression of *I*_K(M)_ observed in the mHippoE-14 cells appears to be independent of the activity of surface tyrosine kinases.

Epileptic seizures are known to be a frequent symptom in patients with glioma [47,48] and tumor-related seizures are strongly linked to the activity of Na_V_ channels in glioma cells [47,49]. Of importance, certain antiepileptic drugs known to block Na_V_ channels have recently been demonstrated to be efficacious in prolonging the survival of patients with malignant gliomas [48,50,51]. PER has previously been reported to have beneficial effects on glioma-associated epilepsy [16,17,18,19]. It is thus tempting to speculate that, despite its blockade of AMPA receptors, PER may be beneficial for tumor-related seizures, since it is capable of blocking the transient and persistent components of *I*_Na_ seen in glioma cells.

The findings of this study indicate that PER-induced blockade of *I*_K(M)_ and *I*_Na_ may synergistically affect the functional activities of central neurons such as hippocampal and motor neurons. Therefore, caution should be taken with regards to its growing use as an antagonist of AMPA receptors [1,7,44,52]. In addition, to what extent the adverse effects (e.g., giddiness) caused by PER [4,53,54] can be relieved by ezogabine, levetiracetam or zinc remains to be determined, because these agents can activate *I*_K(M)_ [40,55,56,57,58]. 

Interestingly, increasing evidence has shown that the gain of function in potassium channel variants is actually associated with epilepsy, including KCNQ2 and KCNQ3 [59]. The predominant hypothesis is that these variants dampen the excitability of interneurons [60] and KNCQ2/3 channels have been shown to be expressed in cortical interneurons [61]. An alternative explanation is that the left-shifted KCNQ2/3 channel gain of function hyperpolarizes the axon initial segment, thereby decreasing the steady-state inactivation of sodium channels [59]. Further clinical investigations are warranted to investigate the potential synergistic effects of PER on the modulation of *I*_K(M)_ and *I*_Na_, in addition to blockade of AMPA receptors on the wide-spectrum of epileptic and neurological disorders. Alternatively, most of established anti-epileptic drugs (e.g., phenytoin, carbamazepine, lamotrigine or others) are Na_V_ channel blockers. However, whether they could perturb *I*_K(M)_ or *I*_K(DR)_ in central neurons is largely unknown and remains to be further investigated.

There are limitations in our study. Ideally, it would be interesting to evaluate the effect of PER on ionic currents, when the cells express AMPA receptors on their surface membranes and to investigate the simultaneous actions of all the ion channels of interest. However, under those experimental conditions, the overlapping by the PER binding to AMPA receptors, and/or by its simultaneous interactions with different types of ionic currents (e.g., [*I*_Na_] and [*I*_K(M)_]) would potentially become too exaggerated to be accurately studied during the recordings. 

Nonetheless, our studies indeed highlight the notion that, in addition to the antagonistic action on AMPA receptors, the PER molecules per se are capable of interacting directly with membrane ionic channels to modify the amplitude and gating of ionic currents. Since the modifications of *I*_Na_ and *I*_K(M)_ significantly regulate the behaviors of electrically excitable cells, such actions on these currents are particularly of clinical, pharmacological or toxicological relevance. 

## Abbreviations

AMPA	α-amino-3-hydroxy-5-methyl-4-isoxazolepropionic acid
DMEM	Dulbecco’s modified Eagle’s medium
FBS	fetal bovine serum
*I-V*	current versus voltage
IK_Ca_ channel	intermediate-conductance Ca^2+^-activated K^+^ channel
*I*_K(DR)_	delayed-rectifier K^+^ current
*I*_K(M)_	M-type K^+^ current
*I*_Na_	voltage-gated Na^+^ current
K_M_ channel	M-type K^+^ channel
K_V_ channel	voltage-gated K^+^ channel
Na_V_ channel	voltage-gated Na^+^ channel
PER	perampanel
NBQX disodium salt	2,3-dioxo-6-nitro-1,2,3,4-tetrahydrobenzo[*f*]quinozaline-7-sulfonamide disodium salt
QA	L-quisqualic acid
τ_deact_	deactivation time constant
TEA	tetraethylammonium chloride
Tef	tefluthrin
TTX	tetrodotoxin
